# (*E*)-2,2′-[3-(2-Nitro­phen­yl)prop-2-ene-1,1-di­yl]bis­(3-hy­droxy-5,5-dimethyl­cyclo­hex-2-en-1-one)

**DOI:** 10.1107/S1600536811043686

**Published:** 2011-11-02

**Authors:** Joo Hwan Cha, Young Hee Kim, Sun-Joon Min, Yong Seo Cho, Jae Kyun Lee

**Affiliations:** a Advanced Analysis Center, Korea Institute of Science and Technology, Hwarangro 14-gil, Seongbuk-gu, Seoul 136-791, Republic of Korea; b Center for Neuro-Medicine, Korea Institute of Science and Technology, Hwarangro 14-gil, Seongbuk-gu, Seoul 136-791, Republic of Korea

## Abstract

In the title compound, C_25_H_29_NO_6_, each of the cyclo­hexenone rings adopts a half-chair conformation. Each of the pairs of hy­droxy and carbonyl O atoms are oriented to allow for the formation of intra­molecular O—H⋯O hydrogen bonds, which are typical of xanthene derivatives. The nitro group is rotationally disordered over two orientations in a 0.544 (6):0.456 (6) ratio. In the crystal, weak inter­molecualr C—H⋯O hydrogen bonds link mol­ecules into layers parallel to the *ab* plane.

## Related literature

For related structures of xanthenes, see: Bolte *et al.* (2001 ▶); Palakshi Reddy *et al.* (2010 ▶); Zhu *et al.* (2011 ▶); Cha *et al.* (2011 ▶).
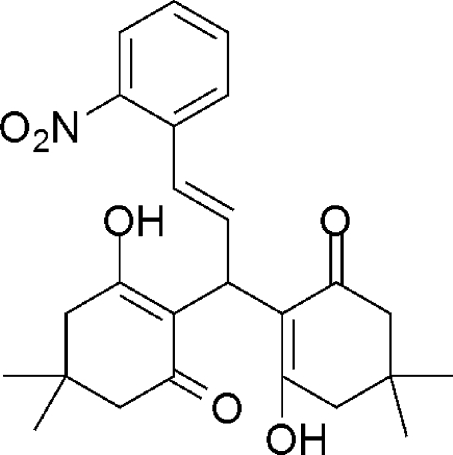


## Experimental

### 

#### Crystal data

C_25_H_29_NO_6_
*M* *_r_* = 439.51
Triclinic, 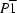

*a* = 9.8306 (14) Å
*b* = 11.0841 (14) Å
*c* = 11.9602 (13) Å
α = 69.601 (3)°
β = 79.867 (4)°
γ = 72.588 (4)°
*V* = 1161.8 (3) Å^3^
*Z* = 2
Mo *K*α radiation
μ = 0.09 mm^−1^
*T* = 298 K
0.20 × 0.10 × 0.10 mm

#### Data collection

Rigaku R-AXIS RAPID diffractometer
Absorption correction: multi-scan (*ABSCOR*; Rigaku, 1995 ▶) *T*_min_ = 0.657, *T*_max_ = 0.991
9400 measured reflections
4194 independent reflections
1687 reflections with *F* ^2^ > 2σ(*F* ^2^)
*R*_int_ = 0.062

#### Refinement

*R*[*F* ^2^ > 2σ(*F* ^2^)] = 0.064
*wR*(*F* ^2^) = 0.191
*S* = 0.99
4194 reflections
313 parameters
336 restraints
H-atom parameters constrained
Δρ_max_ = 0.38 e Å^−3^
Δρ_min_ = −0.45 e Å^−3^

### 

Data collection: *RAPID-AUTO* (Rigaku, 2006 ▶); cell refinement: *RAPID-AUTO*; data reduction: *RAPID-AUTO*; program(s) used to solve structure: *IL MILIONE* (Burla *et al.*, 2007 ▶); program(s) used to refine structure: *SHELXL97* (Sheldrick, 2008 ▶); molecular graphics: *CrystalStructure* (Rigaku, 2010 ▶); software used to prepare material for publication: *CrystalStructure*.

## Supplementary Material

Crystal structure: contains datablock(s) global, I. DOI: 10.1107/S1600536811043686/cv5170sup1.cif

Structure factors: contains datablock(s) I. DOI: 10.1107/S1600536811043686/cv5170Isup2.hkl

Supplementary material file. DOI: 10.1107/S1600536811043686/cv5170Isup3.cml

Additional supplementary materials:  crystallographic information; 3D view; checkCIF report

## Figures and Tables

**Table 1:** Hydrogen-bond geometry (Å, °)

*D*—H⋯*A*	*D*—H	H⋯*A*	*D*⋯*A*	*D*—H⋯*A*
O2—H2⋯O4	0.82	1.82	2.617 (4)	165
O3—H3*A*⋯O1	0.82	1.83	2.611 (4)	158
C16—H16*C*⋯O6^i^	0.96	2.56	3.449 (11)	155
C20—H20*A*⋯O5^ii^	0.97	2.58	3.509 (12)	161
C22—H22*A*⋯O4^iii^	0.97	2.58	3.530 (5)	165
C22—H22*B*⋯O6*a*^ii^	0.97	2.54	3.342 (9)	140

Symmetry codes: (i) 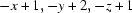; (ii) 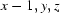; (iii) 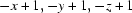.

**Fig. 1.**
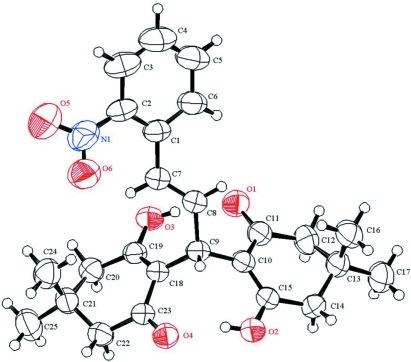
The molecular structure of (I) showing the atomic numbering and 50% probability displacement ellipsoid. Only major part of the disordered nitro group is shown.

## supplementary crystallographic information

### Comment

As a part of our ongoing study of the substituent effect on the solid state structures of Xanthene derivatives (Cha *et al.*, 2011) we present here the crystal structure of the title compound (I) (Fig. 1).

In (I) (Fig. 1), the bond lengths and angles are normal and correspond to those observed in related structures (Bolte *et al.*, 2001; Palakshi Reddy *et al.*, 2010; Zhu *et al.*, 2011; Cha *et al.*, 2011). In the title compound, the dihedral angle between the 2-nitrobenzene and 3-hydroxy-5,5-dimethylcyclohex-2-enone rings are 69.6 (4)° and 42.1 (1)°, respectively. All two cyclohexenone rings in (Fig.1) display half-chair conformation. The hydroxy and carbonyl O atoms face each other and are orientated to allow for the formation of two intramolecular O—H···O hydrogen bonds (Table 1) which are typical of xanthene derivatives. The nitro group is rotationally disordered over two orientations in a ratio 0.544 (6):0.456 (6).

In the crystal, weak intermolecular C—H···O hydrogen bonds (Table 1) link molecules into layers parallel to the *ab* plane.

### Experimental

To solution of 1,3-cyclohexanedione (4.61 mmol), 2-nitrocinnamaldehyde (1.84 mmol) and 4° MS was added catalytic amounts of *L*-proline (0.47 mmol) in under nitrogen atmosphere. After stirring for 5 h, The anhydrous ethyl acetate (0.5 ml) was added to a reaction mixture and the solution was stirred for 3 days. The reaction mixture was filtered through pad of celite to remove MS and concentrated. The residue oil was purified by flash column chromatography to afford product which was recrystallized from ethanol to give crystals suitable for X-ray analysis.

### Refinement

All H atoms were positioned geometrically and refined using a riding model with C—H = 0.93–0.98 Å and *U*iso(H) = 1.2 or 1.5*U*eq(C). Rotating group model was applied for the methyl groups. The nitro group was refined as disordered over two orientations.

### Crystal data

**Table:**

C_25_H_29_NO_6_	*Z* = 2
*M**_r_* = 439.51	*F*(000) = 468.00
Triclinic, *P*1	*D*_x_ = 1.256 Mg m^−^^3^
Hall symbol: -P 1	Mo *K*α radiation, λ = 0.71075 Å
*a* = 9.8306 (14) Å	Cell parameters from 5247 reflections
*b* = 11.0841 (14) Å	θ = 3.1–27.5°
*c* = 11.9602 (13) Å	µ = 0.09 mm^−^^1^
α = 69.601 (3)°	*T* = 298 K
β = 79.867 (4)°	Block, colourless
γ = 72.588 (4)°	0.20 × 0.10 × 0.10 mm
*V* = 1161.8 (3) Å^3^	

### Data collection

**Table:**

Rigaku R-AXIS RAPID diffractometer	1687 reflections with *F*^2^ > 2σ(*F*^2^)
Detector resolution: 10.000 pixels mm^-1^	*R*_int_ = 0.062
ω scans	θ_max_ = 25.3°
Absorption correction: multi-scan (*ABSCOR*; Rigaku, 1995)	*h* = −11→11
*T*_min_ = 0.657, *T*_max_ = 0.991	*k* = −13→13
9400 measured reflections	*l* = −14→13
4194 independent reflections	

### Refinement

**Table:**

Refinement on *F*^2^	Secondary atom site location: difference Fourier map
*R*[*F*^2^ > 2σ(*F*^2^)] = 0.064	Hydrogen site location: inferred from neighbouring sites
*wR*(*F*^2^) = 0.191	H-atom parameters constrained
*S* = 0.99	*w* = 1/[σ^2^(*F*_o_^2^) + (0.0813*P*)^2^] where *P* = (*F*_o_^2^ + 2*F*_c_^2^)/3
4194 reflections	(Δ/σ)_max_ < 0.001
313 parameters	Δρ_max_ = 0.38 e Å^−^^3^
336 restraints	Δρ_min_ = −0.45 e Å^−^^3^
Primary atom site location: structure-invariant direct methods	

### Special details

**Table:**

Geometry. All e.s.d.'s (except the e.s.d. in the dihedral angle between two l.s. planes) are estimated using the full covariance matrix. The cell e.s.d.'s are taken into account individually in the estimation of e.s.d.'s in distances, angles and torsion angles; correlations between e.s.d.'s in cell parameters are only used when they are defined by crystal symmetry. An approximate (isotropic) treatment of cell e.s.d.'s is used for estimating e.s.d.'s involving l.s. planes.
Refinement. Refinement was performed using all reflections. The weighted *R*-factor (*wR*) and goodness of fit (*S*) are based on *F*^2^. *R*-factor (gt) are based on *F*. The threshold expression of *F*^2^ > 2.0 σ(*F*^2^) is used only for calculating *R*-factor (gt).

### Fractional atomic coordinates and isotropic or equivalent isotropic displacement parameters (Å^2^)

**Table:**

	*x*	*y*	*z*	*U*_iso_*/*U*_eq_	Occ. (<1)
O1	0.2599 (4)	0.9448 (3)	0.8416 (3)	0.0738 (9)	
O2	0.2544 (4)	0.9495 (3)	0.4491 (2)	0.0659 (8)	
O3	0.3523 (3)	0.6854 (3)	0.9110 (2)	0.0645 (8)	
O4	0.3900 (3)	0.6966 (3)	0.5114 (2)	0.0673 (9)	
O5a	0.8470 (8)	0.5275 (8)	0.8424 (8)	0.112 (3)	0.544 (6)
O5	1.0123 (10)	0.4996 (9)	0.8776 (5)	0.138 (3)	0.456 (6)
O6	0.8873 (10)	0.5826 (9)	0.7438 (8)	0.105 (3)	0.456 (6)
O6a	1.0410 (9)	0.5912 (8)	0.7656 (7)	0.131 (3)	0.544 (6)
N1	0.9230 (6)	0.6001 (6)	0.8254 (5)	0.0982 (14)	
C1	0.7340 (5)	0.8011 (5)	0.8334 (4)	0.0613 (10)	
C2	0.8713 (5)	0.7226 (5)	0.8563 (4)	0.0713 (11)	
C3	0.9652 (6)	0.7558 (6)	0.9076 (5)	0.0960 (15)	
C4	0.9227 (7)	0.8681 (6)	0.9389 (5)	0.0977 (16)	
C5	0.7895 (7)	0.9491 (6)	0.9180 (4)	0.0873 (14)	
C6	0.6967 (6)	0.9166 (5)	0.8660 (4)	0.0736 (12)	
C7	0.6299 (5)	0.7701 (4)	0.7807 (4)	0.0585 (10)	
C8	0.5278 (5)	0.8605 (4)	0.7170 (3)	0.0572 (10)	
C9	0.4195 (5)	0.8353 (4)	0.6577 (3)	0.0515 (9)	
C10	0.2823 (4)	0.9502 (4)	0.6418 (3)	0.0520 (10)	
C11	0.2202 (5)	1.0022 (4)	0.7372 (4)	0.0592 (10)	
C12	0.0991 (5)	1.1265 (4)	0.7155 (4)	0.0715 (12)	
C13	0.0902 (5)	1.2149 (4)	0.5852 (4)	0.0638 (11)	
C14	0.0981 (5)	1.1248 (4)	0.5111 (4)	0.0641 (11)	
C15	0.2178 (5)	1.0031 (4)	0.5369 (4)	0.0545 (10)	
C16	0.2137 (6)	1.2809 (5)	0.5453 (4)	0.0798 (13)	
C17	−0.0503 (5)	1.3220 (5)	0.5690 (5)	0.0851 (14)	
C18	0.3973 (4)	0.6970 (4)	0.7065 (3)	0.0487 (9)	
C19	0.3753 (4)	0.6294 (4)	0.8266 (3)	0.0520 (10)	
C20	0.3741 (5)	0.4871 (4)	0.8725 (4)	0.0613 (11)	
C21	0.4418 (5)	0.4095 (4)	0.7843 (4)	0.0617 (11)	
C22	0.3813 (5)	0.4934 (4)	0.6638 (3)	0.0624 (11)	
C23	0.3928 (5)	0.6342 (4)	0.6216 (4)	0.0543 (10)	
C24	0.6045 (5)	0.3842 (5)	0.7714 (4)	0.0799 (13)	
C25	0.4058 (6)	0.2761 (4)	0.8278 (4)	0.0862 (14)	
H2	0.3076	0.8746	0.4732	0.0791*	
H3	1.0571	0.7009	0.9204	0.1152*	
H3A	0.3452	0.7655	0.8804	0.0774*	
H4	0.9847	0.8899	0.9747	0.1172*	
H5	0.7607	1.0269	0.9388	0.1048*	
H6	0.6059	0.9738	0.8523	0.0883*	
H7	0.6365	0.6809	0.7931	0.0702*	
H8	0.5213	0.9489	0.7074	0.0686*	
H9	0.4624	0.8426	0.5759	0.0618*	
H12A	0.1087	1.1783	0.7630	0.0858*	
H12B	0.0099	1.1010	0.7436	0.0858*	
H14A	0.0090	1.0990	0.5261	0.0769*	
H14B	0.1079	1.1751	0.4269	0.0769*	
H16A	0.3028	1.2136	0.5566	0.0957*	
H16B	0.2067	1.3379	0.5920	0.0957*	
H16C	0.2096	1.3328	0.4623	0.0957*	
H17A	−0.0528	1.3823	0.6111	0.1021*	
H17B	−0.1284	1.2813	0.6001	0.1021*	
H17C	−0.0585	1.3702	0.4854	0.1021*	
H20A	0.2759	0.4815	0.8944	0.0735*	
H20B	0.4247	0.4447	0.9447	0.0735*	
H22A	0.4313	0.4511	0.6041	0.0749*	
H22B	0.2813	0.4942	0.6698	0.0749*	
H24A	0.6449	0.3322	0.7184	0.0959*	
H24B	0.6413	0.3368	0.8484	0.0959*	
H24C	0.6295	0.4678	0.7393	0.0959*	
H25A	0.4466	0.2235	0.9025	0.1035*	
H25B	0.4442	0.2304	0.7697	0.1035*	
H25C	0.3038	0.2899	0.8388	0.1035*	

### Atomic displacement parameters (Å^2^)

**Table:**

	*U*^11^	*U*^22^	*U*^33^	*U*^12^	*U*^13^	*U*^23^
O1	0.089 (3)	0.073 (2)	0.0583 (16)	−0.0081 (17)	−0.0155 (15)	−0.0260 (14)
O2	0.080 (3)	0.0581 (18)	0.0631 (16)	−0.0145 (16)	−0.0197 (14)	−0.0191 (13)
O3	0.077 (2)	0.0698 (18)	0.0545 (14)	−0.0182 (15)	−0.0097 (13)	−0.0274 (13)
O4	0.089 (3)	0.0627 (18)	0.0494 (15)	−0.0097 (16)	−0.0154 (14)	−0.0203 (13)
O5a	0.087 (5)	0.107 (5)	0.152 (5)	−0.002 (4)	−0.028 (5)	−0.065 (4)
O5	0.111 (6)	0.122 (5)	0.174 (6)	0.009 (5)	−0.060 (5)	−0.047 (5)
O6	0.092 (5)	0.112 (5)	0.122 (5)	−0.004 (4)	−0.029 (5)	−0.058 (4)
O6a	0.094 (5)	0.141 (5)	0.130 (5)	0.007 (4)	0.002 (4)	−0.044 (4)
N1	0.069 (3)	0.109 (4)	0.116 (3)	0.001 (3)	−0.028 (3)	−0.045 (3)
C1	0.061 (3)	0.072 (3)	0.059 (2)	−0.027 (2)	−0.0112 (19)	−0.0192 (19)
C2	0.056 (3)	0.095 (3)	0.071 (3)	−0.025 (3)	−0.016 (2)	−0.027 (3)
C3	0.065 (4)	0.134 (5)	0.104 (4)	−0.036 (3)	−0.023 (3)	−0.038 (3)
C4	0.092 (4)	0.131 (5)	0.098 (4)	−0.058 (4)	−0.020 (3)	−0.037 (3)
C5	0.102 (4)	0.100 (4)	0.085 (3)	−0.050 (3)	−0.014 (3)	−0.035 (3)
C6	0.082 (4)	0.081 (3)	0.070 (3)	−0.031 (3)	−0.016 (3)	−0.027 (3)
C7	0.060 (3)	0.058 (3)	0.064 (3)	−0.016 (2)	−0.0126 (19)	−0.0224 (19)
C8	0.060 (3)	0.056 (3)	0.062 (3)	−0.017 (2)	−0.0105 (19)	−0.0217 (18)
C9	0.053 (3)	0.055 (2)	0.0522 (19)	−0.0146 (18)	−0.0094 (17)	−0.0205 (16)
C10	0.051 (3)	0.049 (3)	0.063 (2)	−0.0131 (18)	−0.0088 (18)	−0.0229 (17)
C11	0.057 (3)	0.057 (3)	0.067 (3)	−0.013 (2)	−0.008 (2)	−0.0240 (19)
C12	0.069 (3)	0.060 (3)	0.082 (3)	−0.006 (2)	−0.001 (2)	−0.030 (2)
C13	0.054 (3)	0.056 (3)	0.081 (3)	−0.0065 (19)	−0.014 (2)	−0.0239 (18)
C14	0.061 (3)	0.056 (3)	0.076 (3)	−0.013 (2)	−0.023 (2)	−0.0152 (19)
C15	0.058 (3)	0.047 (3)	0.064 (2)	−0.0163 (19)	−0.0136 (18)	−0.0179 (18)
C16	0.077 (4)	0.066 (3)	0.104 (3)	−0.025 (3)	−0.014 (3)	−0.028 (3)
C17	0.073 (4)	0.069 (3)	0.110 (4)	−0.003 (3)	−0.018 (3)	−0.031 (3)
C18	0.050 (3)	0.048 (2)	0.0515 (19)	−0.0115 (18)	−0.0107 (17)	−0.0183 (16)
C19	0.049 (3)	0.056 (3)	0.056 (2)	−0.0114 (19)	−0.0090 (17)	−0.0224 (17)
C20	0.064 (3)	0.060 (3)	0.060 (2)	−0.017 (2)	−0.0070 (19)	−0.0177 (18)
C21	0.071 (3)	0.049 (2)	0.064 (2)	−0.012 (2)	−0.0081 (19)	−0.0177 (17)
C22	0.074 (3)	0.060 (3)	0.059 (2)	−0.018 (3)	−0.009 (2)	−0.0244 (18)
C23	0.058 (3)	0.053 (3)	0.058 (2)	−0.0104 (19)	−0.0155 (18)	−0.0219 (17)
C24	0.079 (4)	0.075 (3)	0.078 (3)	−0.006 (3)	−0.008 (3)	−0.026 (3)
C25	0.111 (4)	0.061 (3)	0.087 (3)	−0.023 (3)	−0.006 (3)	−0.022 (3)

### Geometric parameters (Å, °)

**Table:**

O1—C11	1.259 (5)	C21—C24	1.528 (7)
O2—C15	1.327 (6)	C21—C25	1.514 (7)
O3—C19	1.315 (6)	C22—C23	1.497 (6)
O4—C23	1.257 (4)	O2—H2	0.820
O5a—N1	1.199 (12)	O3—H3A	0.820
O5—N1	1.235 (9)	C3—H3	0.930
O6—N1	1.186 (14)	C4—H4	0.930
O6a—N1	1.247 (10)	C5—H5	0.930
N1—C2	1.453 (9)	C6—H6	0.930
C1—C2	1.384 (6)	C7—H7	0.930
C1—C6	1.394 (8)	C8—H8	0.930
C1—C7	1.467 (8)	C9—H9	0.980
C2—C3	1.387 (10)	C12—H12A	0.970
C3—C4	1.350 (10)	C12—H12B	0.970
C4—C5	1.355 (8)	C14—H14A	0.970
C5—C6	1.379 (10)	C14—H14B	0.970
C7—C8	1.314 (5)	C16—H16A	0.960
C8—C9	1.515 (7)	C16—H16B	0.960
C9—C10	1.540 (5)	C16—H16C	0.960
C9—C18	1.507 (6)	C17—H17A	0.960
C10—C11	1.419 (6)	C17—H17B	0.960
C10—C15	1.365 (6)	C17—H17C	0.960
C11—C12	1.503 (5)	C20—H20A	0.970
C12—C13	1.526 (6)	C20—H20B	0.970
C13—C14	1.529 (8)	C22—H22A	0.970
C13—C16	1.524 (8)	C22—H22B	0.970
C13—C17	1.519 (6)	C24—H24A	0.960
C14—C15	1.478 (5)	C24—H24B	0.960
C18—C19	1.381 (5)	C24—H24C	0.960
C18—C23	1.430 (7)	C25—H25A	0.960
C19—C20	1.482 (6)	C25—H25B	0.960
C20—C21	1.529 (6)	C25—H25C	0.960
C21—C22	1.528 (5)		
			
O1···O3	2.611 (4)	H22A···H25A	3.5787
O1···C8	2.855 (5)	H22A···H25B	2.5392
O1···C9	2.878 (5)	H22A···H25C	3.0413
O1···C15	3.549 (5)	H22B···H24A	3.5349
O1···C18	3.476 (5)	H22B···H24C	3.5669
O1···C19	3.396 (5)	H22B···H25A	3.4987
O2···O4	2.617 (4)	H22B···H25B	2.8245
O2···C9	2.908 (5)	H22B···H25C	2.4356
O2···C18	3.528 (4)	H24A···H25A	2.8542
O2···C23	3.381 (4)	H24A···H25B	2.4418
O3···C7	3.126 (5)	H24A···H25C	3.5079
O3···C8	3.095 (5)	H24B···H25A	2.4687
O3···C9	2.970 (4)	H24B···H25B	2.9684
O3···C10	3.527 (4)	H24B···H25C	3.5347
O3···C11	3.387 (4)	H24C···H25A	3.5173
O4···C9	2.799 (6)	H24C···H25B	3.5131
O4···C10	3.485 (6)	O1···H4^i^	3.0030
O4···C15	3.406 (5)	O1···H4^ii^	3.5032
O4···C19	3.564 (5)	O1···H5^ii^	2.7182
O5a···C1	2.871 (10)	O2···H6^iii^	3.5246
O5a···C3	3.438 (13)	O2···H8^iii^	2.8634
O5a···C7	2.840 (8)	O2···H14A^vii^	2.7444
O5a···C24	3.584 (12)	O2···H17B^vii^	3.4098
O5···C1	3.569 (9)	O3···H3^i^	2.8403
O5···C3	2.877 (13)	O3···H20B^viii^	2.7509
O6···C1	2.889 (11)	O3···H24B^viii^	2.8118
O6···C3	3.485 (15)	O4···H16A^iii^	3.3391
O6···C7	2.832 (10)	O4···H17B^vii^	3.0090
O6a···C1	3.380 (9)	O4···H17C^vii^	3.5422
O6a···C3	2.769 (12)	O4···H22A^iv^	2.5841
N1···C7	2.954 (7)	O4···H24A^iv^	2.9628
C1···C4	2.808 (10)	O4···H25B^iv^	3.4027
C2···C5	2.714 (9)	O5a···H3^v^	3.1383
C3···C6	2.724 (7)	O5a···H16C^iii^	3.4996
C6···C8	2.958 (8)	O5a···H17A^ix^	3.5217
C7···C18	2.998 (7)	O5a···H20A^viii^	3.1464
C7···C19	3.201 (7)	O5a···H20B^viii^	3.3734
C8···C11	2.973 (6)	O5···H3^v^	2.7963
C8···C19	3.129 (7)	O5···H20A^vi^	2.5768
C10···C13	2.896 (5)	O5···H20A^viii^	3.5819
C10···C16	3.327 (6)	O5···H22B^vi^	3.2977
C10···C19	3.413 (5)	O5···H25C^vi^	3.1827
C10···C23	3.424 (6)	O6···H14B^iii^	2.7574
C11···C14	2.865 (6)	O6···H16C^iii^	2.5568
C11···C16	3.131 (6)	O6···H17A^ix^	3.0218
C11···C18	3.425 (6)	O6···H17C^iii^	2.9193
C12···C15	2.828 (7)	O6a···H14B^iii^	2.9855
C15···C16	3.103 (7)	O6a···H17C^iii^	2.8625
C15···C18	3.409 (5)	O6a···H20A^vi^	2.7289
C18···C21	2.908 (6)	O6a···H22B^vi^	2.5375
C18···C24	3.350 (6)	O6a···H25C^vi^	3.4915
C19···C22	2.832 (7)	N1···H14B^iii^	3.1675
C19···C24	3.156 (6)	N1···H20A^vi^	3.4593
C20···C23	2.877 (5)	N1···H20A^viii^	3.5375
C23···C24	3.101 (5)	C1···H14B^iii^	3.1847
O1···C4^i^	3.564 (8)	C1···H20B^viii^	3.5984
O1···C5^ii^	3.378 (7)	C1···H25A^viii^	3.3048
O2···C1^iii^	3.552 (4)	C2···H14B^iii^	3.1649
O2···C6^iii^	3.540 (5)	C2···H20A^viii^	3.4410
O2···C8^iii^	3.355 (6)	C4···H4^x^	3.5792
O4···C22^iv^	3.530 (5)	C4···H12A^ii^	3.3911
O5···O5^v^	2.898 (10)	C4···H12B^vi^	3.0445
O5···N1^v^	3.447 (9)	C4···H25C^viii^	3.4784
O5···C2^v^	3.411 (8)	C5···H12B^vi^	3.1658
O5···C3^v^	3.068 (9)	C5···H25A^viii^	3.4201
O5···C20^vi^	3.509 (12)	C5···H25C^viii^	3.3856
O6···C16^iii^	3.449 (11)	C6···H25A^viii^	2.9999
O6a···C20^vi^	3.446 (10)	C6···H25B^xi^	3.5538
O6a···C22^vi^	3.342 (9)	C6···H25C^viii^	3.4738
N1···O5^v^	3.447 (9)	C7···H14B^iii^	3.2878
C1···O2^iii^	3.552 (4)	C7···H16C^iii^	3.4424
C2···O5^v^	3.411 (8)	C7···H20B^viii^	3.3819
C3···O5^v^	3.068 (9)	C9···H16A^iii^	3.4547
C4···O1^vi^	3.564 (8)	C11···H4^i^	3.4528
C5···O1^ii^	3.378 (7)	C12···H4^i^	3.5531
C6···O2^iii^	3.540 (5)	C14···H14A^vii^	3.1557
C8···O2^iii^	3.355 (6)	C15···H8^iii^	3.5347
C16···O6^iii^	3.449 (11)	C15···H14A^vii^	3.0848
C20···O5^i^	3.509 (12)	C16···H9^iii^	3.3562
C20···O6a^i^	3.446 (10)	C16···H17A^xii^	3.5465
C22···O4^iv^	3.530 (5)	C16···H22A^xi^	3.5359
C22···O6a^i^	3.342 (9)	C16···H22B^xi^	3.4629
O1···H3A	1.8342	C17···H16B^xii^	3.5769
O1···H6	3.5399	C17···H16C^xii^	3.5852
O1···H8	2.7844	C17···H17A^xii^	3.5645
O1···H12A	2.5070	C17···H22B^vii^	3.5945
O1···H12B	2.7319	C17···H24A^xiii^	3.1998
O2···H9	2.5051	C19···H3^i^	3.0945
O2···H14A	2.7026	C19···H20B^viii^	3.3743
O2···H14B	2.4319	C20···H3^i^	3.3994
O3···H7	2.8942	C20···H20B^viii^	3.5822
O3···H20A	2.6639	C22···H16B^xiv^	3.1583
O3···H20B	2.4504	C22···H17C^vii^	3.5747
O4···H2	1.8174	C22···H22A^iv^	3.3566
O4···H9	2.3403	C23···H22A^iv^	3.2143
O4···H22A	2.4877	C24···H17A^ix^	3.5690
O4···H22B	2.7420	C24···H17B^ix^	3.2147
O5a···H7	2.2686	C25···H6^xiv^	3.2801
O5a···H24B	3.3115	C25···H16B^xiv^	3.4712
O5a···H24C	2.9790	H2···H8^iii^	3.0113
O5···H3	2.6267	H2···H14A^vii^	3.0366
O6···H7	2.4376	H2···H17B^vii^	3.2064
O6···H24C	3.1729	H3···O3^vi^	2.8403
O6a···H3	2.5921	H3···O5a^v^	3.1383
N1···H3	2.5641	H3···O5^v^	2.7963
N1···H7	2.7408	H3···C19^vi^	3.0945
C1···H3	3.2634	H3···C20^vi^	3.3994
C1···H5	3.2605	H3···H3A^vi^	3.0433
C1···H8	2.5813	H3···H20A^vi^	2.7926
C2···H4	3.2112	H3A···H3^i^	3.0433
C2···H6	3.1880	H3A···H4^i^	3.5339
C2···H7	2.7579	H3A···H5^ii^	3.5069
C3···H5	3.1800	H3A···H20B^viii^	3.1680
C4···H6	3.2011	H3A···H24B^viii^	3.0533
C5···H3	3.1824	H4···O1^vi^	3.0030
C6···H4	3.2121	H4···O1^ii^	3.5032
C6···H7	3.2603	H4···C4^x^	3.5792
C6···H8	2.6468	H4···C11^vi^	3.4528
C7···H3A	2.8506	H4···C12^vi^	3.5531
C7···H6	2.6167	H4···H3A^vi^	3.5339
C7···H9	2.9336	H4···H4^x^	2.8184
C7···H24C	3.5544	H4···H5^x^	3.3384
C8···H3A	2.5711	H4···H12A^ii^	3.0006
C8···H6	2.6862	H4···H12B^vi^	2.9619
C9···H2	2.4829	H4···H12B^ii^	3.4153
C9···H3A	2.5386	H5···O1^ii^	2.7182
C9···H7	2.6835	H5···H3A^ii^	3.5069
C10···H2	2.3880	H5···H4^x^	3.3384
C10···H3A	2.9169	H5···H12B^vi^	3.1592
C10···H8	2.6008	H5···H24A^xi^	3.4969
C10···H12A	3.2426	H5···H24B^xi^	3.1287
C10···H12B	3.0073	H5···H25A^xi^	3.2026
C10···H14A	2.9856	H6···O2^iii^	3.5246
C10···H14B	3.2068	H6···C25^xi^	3.2801
C10···H16A	2.7945	H6···H25A^xi^	2.9513
C11···H3A	2.6463	H6···H25A^viii^	3.0533
C11···H8	2.8233	H6···H25B^xi^	2.7351
C11···H9	3.2808	H7···H14B^iii^	3.5952
C11···H14A	3.2339	H7···H16C^iii^	3.1934
C11···H16A	2.7944	H7···H20B^viii^	2.9886
C11···H16B	3.4867	H8···O2^iii^	2.8634
C12···H14A	2.7122	H8···C15^iii^	3.5347
C12···H14B	3.2924	H8···H2^iii^	3.0113
C12···H16A	2.6627	H8···H9^iii^	3.3798
C12···H16B	2.7033	H8···H25B^xi^	3.2971
C12···H16C	3.3288	H9···C16^iii^	3.3562
C12···H17A	2.7355	H9···H8^iii^	3.3798
C12···H17B	2.6436	H9···H9^iii^	3.5603
C12···H17C	3.3293	H9···H16A^iii^	2.6017
C14···H2	3.0444	H9···H16C^iii^	3.2761
C14···H12A	3.2916	H12A···C4^ii^	3.3911
C14···H12B	2.7082	H12A···H4^ii^	3.0006
C14···H16A	2.6932	H12A···H25B^xi^	3.5332
C14···H16B	3.3344	H12A···H25C^xi^	2.9740
C14···H16C	2.6837	H12B···C4^i^	3.0445
C14···H17A	3.3171	H12B···C5^i^	3.1658
C14···H17B	2.6918	H12B···H4^i^	2.9619
C14···H17C	2.6349	H12B···H4^ii^	3.4153
C15···H9	2.5344	H12B···H5^i^	3.1592
C15···H12B	3.1990	H14A···O2^vii^	2.7444
C15···H16A	2.7950	H14A···C14^vii^	3.1557
C15···H16C	3.4246	H14A···C15^vii^	3.0848
C16···H12A	2.6131	H14A···H2^vii^	3.0366
C16···H12B	3.3222	H14A···H14A^vii^	2.5426
C16···H14A	3.3289	H14A···H14B^vii^	3.3968
C16···H14B	2.6213	H14B···O6^iii^	2.7574
C16···H17A	2.6225	H14B···O6a^iii^	2.9855
C16···H17B	3.3104	H14B···N1^iii^	3.1675
C16···H17C	2.6927	H14B···C1^iii^	3.1847
C17···H12A	2.7523	H14B···C2^iii^	3.1649
C17···H12B	2.5912	H14B···C7^iii^	3.2878
C17···H14A	2.5751	H14B···H7^iii^	3.5952
C17···H14B	2.7287	H14B···H14A^vii^	3.3968
C17···H16A	3.3107	H16A···O4^iii^	3.3391
C17···H16B	2.6519	H16A···C9^iii^	3.4547
C17···H16C	2.6645	H16A···H9^iii^	2.6017
C18···H2	2.9046	H16A···H22A^xi^	3.4848
C18···H3A	2.3784	H16A···H25B^xi^	3.1936
C18···H7	2.6666	H16B···C17^xii^	3.5769
C18···H8	3.3684	H16B···C22^xi^	3.1583
C18···H20A	3.0331	H16B···C25^xi^	3.4712
C18···H20B	3.2050	H16B···H17A^xii^	3.3232
C18···H22A	3.2670	H16B···H17C^xii^	3.0023
C18···H22B	2.9904	H16B···H22A^xi^	2.8937
C18···H24C	2.8224	H16B···H22B^xi^	2.5559
C19···H7	2.7270	H16B···H25B^xi^	3.1152
C19···H9	3.2664	H16B···H25C^xi^	3.0826
C19···H22B	3.1642	H16C···O5a^iii^	3.4996
C19···H24B	3.4641	H16C···O6^iii^	2.5568
C19···H24C	2.8724	H16C···C7^iii^	3.4424
C20···H3A	3.0466	H16C···C17^xii^	3.5852
C20···H22A	3.3016	H16C···H7^iii^	3.1934
C20···H22B	2.7055	H16C···H9^iii^	3.2761
C20···H24A	3.3430	H16C···H17A^xii^	2.9580
C20···H24B	2.6842	H16C···H17C^xii^	3.4087
C20···H24C	2.7197	H16C···H24C^iii^	3.1993
C20···H25A	2.6991	H17A···O5a^xiii^	3.5217
C20···H25B	3.3242	H17A···O6^xiii^	3.0218
C20···H25C	2.6521	H17A···C16^xii^	3.5465
C22···H20A	2.7423	H17A···C17^xii^	3.5645
C22···H20B	3.2959	H17A···C24^xiii^	3.5690
C22···H24A	2.7098	H17A···H16B^xii^	3.3232
C22···H24B	3.3286	H17A···H16C^xii^	2.9580
C22···H24C	2.6519	H17A···H17A^xii^	3.2813
C22···H25A	3.3247	H17A···H17C^xii^	3.0329
C22···H25B	2.6657	H17A···H24A^xiii^	3.1522
C22···H25C	2.6881	H17A···H24C^xiii^	3.2490
C23···H2	2.6153	H17B···O2^vii^	3.4098
C23···H9	2.4571	H17B···O4^vii^	3.0090
C23···H20A	3.2901	H17B···C24^xiii^	3.2147
C23···H24A	3.4684	H17B···H2^vii^	3.2064
C23···H24C	2.7518	H17B···H22B^vii^	3.5698
C24···H7	3.4968	H17B···H24A^xiii^	2.4454
C24···H20A	3.3332	H17B···H24B^xiii^	3.5206
C24···H20B	2.6149	H17B···H24C^xiii^	3.2810
C24···H22A	2.6186	H17C···O4^vii^	3.5422
C24···H22B	3.3259	H17C···O6^iii^	2.9193
C24···H25A	2.6254	H17C···O6a^iii^	2.8625
C24···H25B	2.6517	H17C···C22^vii^	3.5747
C24···H25C	3.3017	H17C···H16B^xii^	3.0023
C25···H20A	2.5714	H17C···H16C^xii^	3.4087
C25···H20B	2.7563	H17C···H17A^xii^	3.0329
C25···H22A	2.7239	H17C···H17C^xii^	3.5419
C25···H22B	2.6024	H17C···H22B^vii^	2.8316
C25···H24A	2.6166	H20A···O5a^viii^	3.1464
C25···H24B	2.6679	H20A···O5^i^	2.5768
C25···H24C	3.2985	H20A···O5^viii^	3.5819
H2···H9	1.9955	H20A···O6a^i^	2.7289
H2···H14A	3.3585	H20A···N1^i^	3.4593
H2···H14B	3.2310	H20A···N1^viii^	3.5375
H3···H4	2.2832	H20A···C2^viii^	3.4410
H3A···H7	2.8629	H20A···H3^i^	2.7926
H3A···H8	3.0536	H20B···O3^viii^	2.7509
H3A···H9	3.5015	H20B···O5a^viii^	3.3734
H3A···H20A	3.3586	H20B···C1^viii^	3.5984
H3A···H20B	3.2391	H20B···C7^viii^	3.3819
H4···H5	2.2837	H20B···C19^viii^	3.3743
H5···H6	2.2902	H20B···C20^viii^	3.5822
H6···H7	3.4744	H20B···H3A^viii^	3.1680
H6···H8	2.1787	H20B···H7^viii^	2.9886
H7···H8	2.7253	H20B···H20B^viii^	2.8907
H7···H9	3.0610	H22A···O4^iv^	2.5841
H7···H24C	2.6782	H22A···C16^xiv^	3.5359
H8···H9	2.4936	H22A···C22^iv^	3.3566
H8···H16A	3.2218	H22A···C23^iv^	3.2143
H12A···H16A	2.8289	H22A···H16A^xiv^	3.4848
H12A···H16B	2.4577	H22A···H16B^xiv^	2.8937
H12A···H16C	3.5099	H22A···H22A^iv^	2.6484
H12A···H17A	2.6336	H22B···O5^i^	3.2977
H12A···H17B	2.9976	H22B···O6a^i^	2.5375
H12B···H14A	2.6107	H22B···C16^xiv^	3.4629
H12B···H16A	3.5761	H22B···C17^vii^	3.5945
H12B···H16B	3.5225	H22B···H16B^xiv^	2.5559
H12B···H17A	2.8916	H22B···H17B^vii^	3.5698
H12B···H17B	2.3643	H22B···H17C^vii^	2.8316
H12B···H17C	3.4639	H24A···O4^iv^	2.9628
H14A···H16C	3.5119	H24A···C17^ix^	3.1998
H14A···H17A	3.4831	H24A···H5^xiv^	3.4969
H14A···H17B	2.4259	H24A···H17A^ix^	3.1522
H14A···H17C	2.7595	H24A···H17B^ix^	2.4454
H14B···H16A	2.8728	H24B···O3^viii^	2.8118
H14B···H16B	3.5081	H24B···H3A^viii^	3.0533
H14B···H16C	2.4376	H24B···H5^xiv^	3.1287
H14B···H17A	3.5723	H24B···H17B^ix^	3.5206
H14B···H17B	3.0739	H24C···H16C^iii^	3.1993
H14B···H17C	2.5258	H24C···H17A^ix^	3.2490
H16A···H17A	3.5041	H24C···H17B^ix^	3.2810
H16A···H17C	3.5631	H25A···C1^viii^	3.3048
H16B···H17A	2.4337	H25A···C5^viii^	3.4201
H16B···H17B	3.5119	H25A···C6^viii^	2.9999
H16B···H17C	2.9736	H25A···H5^xiv^	3.2026
H16C···H17A	2.8886	H25A···H6^xiv^	2.9513
H16C···H17B	3.5546	H25A···H6^viii^	3.0533
H16C···H17C	2.5226	H25B···O4^iv^	3.4027
H20A···H22B	2.6318	H25B···C6^xiv^	3.5538
H20A···H24B	3.4999	H25B···H6^xiv^	2.7351
H20A···H25A	2.8273	H25B···H8^xiv^	3.2971
H20A···H25B	3.4607	H25B···H12A^xiv^	3.5332
H20A···H25C	2.3657	H25B···H16A^xiv^	3.1936
H20B···H24A	3.4986	H25B···H16B^xiv^	3.1152
H20B···H24B	2.4210	H25C···O5^i^	3.1827
H20B···H24C	2.8781	H25C···O6a^i^	3.4915
H20B···H25A	2.6110	H25C···C4^viii^	3.4784
H20B···H25C	3.0365	H25C···C5^viii^	3.3856
H22A···H24A	2.4715	H25C···C6^viii^	3.4738
H22A···H24B	3.5179	H25C···H12A^xiv^	2.9740
H22A···H24C	2.8237	H25C···H16B^xiv^	3.0826
			
O5a—N1—O6a	125.1 (8)	C4—C3—H3	120.118
O5a—N1—C2	120.9 (6)	C3—C4—H4	120.071
O5—N1—O6	109.5 (8)	C5—C4—H4	120.073
O5—N1—C2	125.8 (7)	C4—C5—H5	119.828
O6—N1—C2	124.8 (6)	C6—C5—H5	119.829
O6a—N1—C2	113.2 (7)	C1—C6—H6	118.852
C2—C1—C6	114.9 (5)	C5—C6—H6	118.851
C2—C1—C7	125.1 (5)	C1—C7—H7	117.990
C6—C1—C7	120.0 (4)	C8—C7—H7	117.987
N1—C2—C1	120.0 (6)	C7—C8—H8	116.681
N1—C2—C3	117.2 (5)	C9—C8—H8	116.681
C1—C2—C3	122.8 (6)	C8—C9—H9	104.325
C2—C3—C4	119.8 (5)	C10—C9—H9	104.329
C3—C4—C5	119.9 (7)	C18—C9—H9	104.333
C4—C5—C6	120.3 (6)	C11—C12—H12A	108.529
C1—C6—C5	122.3 (5)	C11—C12—H12B	108.526
C1—C7—C8	124.0 (5)	C13—C12—H12A	108.527
C7—C8—C9	126.6 (5)	C13—C12—H12B	108.526
C8—C9—C10	110.9 (4)	H12A—C12—H12B	107.525
C8—C9—C18	115.7 (3)	C13—C14—H14A	108.795
C10—C9—C18	115.6 (4)	C13—C14—H14B	108.793
C9—C10—C11	120.0 (4)	C15—C14—H14A	108.798
C9—C10—C15	121.1 (4)	C15—C14—H14B	108.791
C11—C10—C15	118.9 (4)	H14A—C14—H14B	107.676
O1—C11—C10	122.0 (4)	C13—C16—H16A	109.467
O1—C11—C12	118.4 (4)	C13—C16—H16B	109.470
C10—C11—C12	119.6 (4)	C13—C16—H16C	109.467
C11—C12—C13	115.0 (4)	H16A—C16—H16B	109.471
C12—C13—C14	106.9 (4)	H16A—C16—H16C	109.476
C12—C13—C16	110.3 (4)	H16B—C16—H16C	109.476
C12—C13—C17	110.8 (4)	C13—C17—H17A	109.468
C14—C13—C16	110.4 (4)	C13—C17—H17B	109.474
C14—C13—C17	109.3 (5)	C13—C17—H17C	109.466
C16—C13—C17	109.2 (4)	H17A—C17—H17B	109.480
C13—C14—C15	113.8 (4)	H17A—C17—H17C	109.469
O2—C15—C10	123.5 (4)	H17B—C17—H17C	109.471
O2—C15—C14	113.4 (4)	C19—C20—H20A	108.639
C10—C15—C14	123.1 (4)	C19—C20—H20B	108.629
C9—C18—C19	124.7 (4)	C21—C20—H20A	108.630
C9—C18—C23	117.2 (3)	C21—C20—H20B	108.629
C19—C18—C23	118.0 (4)	H20A—C20—H20B	107.589
O3—C19—C18	123.3 (4)	C21—C22—H22A	108.682
O3—C19—C20	113.5 (3)	C21—C22—H22B	108.674
C18—C19—C20	123.2 (4)	C23—C22—H22A	108.681
C19—C20—C21	114.5 (3)	C23—C22—H22B	108.684
C20—C21—C22	107.3 (3)	H22A—C22—H22B	107.597
C20—C21—C24	110.9 (5)	C21—C24—H24A	109.474
C20—C21—C25	110.2 (4)	C21—C24—H24B	109.470
C22—C21—C24	109.8 (4)	C21—C24—H24C	109.471
C22—C21—C25	110.3 (5)	H24A—C24—H24B	109.476
C24—C21—C25	108.3 (4)	H24A—C24—H24C	109.472
C21—C22—C23	114.3 (5)	H24B—C24—H24C	109.464
O4—C23—C18	121.7 (4)	C21—C25—H25A	109.470
O4—C23—C22	118.2 (4)	C21—C25—H25B	109.471
C18—C23—C22	120.0 (3)	C21—C25—H25C	109.468
C15—O2—H2	109.474	H25A—C25—H25B	109.474
C19—O3—H3A	109.472	H25A—C25—H25C	109.471
C2—C3—H3	120.105	H25B—C25—H25C	109.474
			
O5a—N1—C2—C1	−40.8 (8)	C9—C10—C15—O2	10.0 (7)
O5a—N1—C2—C3	139.7 (7)	C9—C10—C15—C14	−171.3 (4)
O5—N1—C2—C1	−150.3 (7)	C11—C10—C15—O2	−169.7 (4)
O5—N1—C2—C3	30.3 (9)	C11—C10—C15—C14	9.1 (7)
O6—N1—C2—C1	31.3 (9)	C15—C10—C11—O1	168.3 (4)
O6—N1—C2—C3	−148.1 (7)	C15—C10—C11—C12	−9.1 (7)
O6a—N1—C2—C1	129.2 (6)	O1—C11—C12—C13	160.1 (4)
O6a—N1—C2—C3	−50.3 (7)	C10—C11—C12—C13	−22.4 (7)
C2—C1—C6—C5	−0.5 (6)	C11—C12—C13—C14	49.9 (6)
C6—C1—C2—N1	−179.5 (3)	C11—C12—C13—C16	−70.2 (5)
C6—C1—C2—C3	−0.1 (6)	C11—C12—C13—C17	168.8 (4)
C2—C1—C7—C8	−151.8 (4)	C12—C13—C14—C15	−49.6 (5)
C7—C1—C2—N1	1.3 (6)	C16—C13—C14—C15	70.3 (4)
C7—C1—C2—C3	−179.2 (3)	C17—C13—C14—C15	−169.6 (3)
C6—C1—C7—C8	29.1 (6)	C13—C14—C15—O2	−158.6 (4)
C7—C1—C6—C5	178.7 (3)	C13—C14—C15—C10	22.5 (6)
N1—C2—C3—C4	−179.6 (4)	C9—C18—C19—O3	9.4 (6)
C1—C2—C3—C4	1.0 (7)	C9—C18—C19—C20	−171.1 (3)
C2—C3—C4—C5	−1.2 (7)	C9—C18—C23—O4	−8.4 (6)
C3—C4—C5—C6	0.6 (7)	C9—C18—C23—C22	175.7 (3)
C4—C5—C6—C1	0.2 (7)	C19—C18—C23—O4	169.0 (4)
C1—C7—C8—C9	178.3 (3)	C19—C18—C23—C22	−6.9 (5)
C7—C8—C9—C10	154.4 (4)	C23—C18—C19—O3	−167.8 (4)
C7—C8—C9—C18	20.2 (6)	C23—C18—C19—C20	11.7 (6)
C8—C9—C10—C11	−45.0 (5)	O3—C19—C20—C21	−163.2 (3)
C8—C9—C10—C15	135.3 (4)	C18—C19—C20—C21	17.3 (6)
C8—C9—C18—C19	48.0 (5)	C19—C20—C21—C22	−47.1 (5)
C8—C9—C18—C23	−134.9 (3)	C19—C20—C21—C24	72.9 (4)
C10—C9—C18—C19	−84.1 (5)	C19—C20—C21—C25	−167.2 (4)
C10—C9—C18—C23	93.1 (4)	C20—C21—C22—C23	51.6 (5)
C18—C9—C10—C11	89.3 (5)	C24—C21—C22—C23	−69.1 (5)
C18—C9—C10—C15	−90.4 (4)	C25—C21—C22—C23	171.6 (3)
C9—C10—C11—O1	−11.4 (7)	C21—C22—C23—O4	157.5 (4)
C9—C10—C11—C12	171.2 (4)	C21—C22—C23—C18	−26.5 (5)

Symmetry codes: (i) *x*−1, *y*, *z*; (ii) −*x*+1, −*y*+2, −*z*+2; (iii) −*x*+1, −*y*+2, −*z*+1; (iv) −*x*+1, −*y*+1, −*z*+1; (v) −*x*+2, −*y*+1, −*z*+2; (vi) *x*+1, *y*, *z*; (vii) −*x*, −*y*+2, −*z*+1; (viii) −*x*+1, −*y*+1, −*z*+2; (ix) *x*+1, *y*−1, *z*; (x) −*x*+2, −*y*+2, −*z*+2; (xi) *x*, *y*+1, *z*; (xii) −*x*, −*y*+3, −*z*+1; (xiii) *x*−1, *y*+1, *z*; (xiv) *x*, *y*−1, *z*.

### Hydrogen-bond geometry (Å, °)

**Table:**

*D*—H···*A*	*D*—H	H···*A*	*D*···*A*	*D*—H···*A*
O2—H2···O4	0.82	1.82	2.617 (4)	165.
O3—H3A···O1	0.82	1.83	2.611 (4)	158.
C16—H16C···O6^iii^	0.96	2.56	3.449 (11)	155.
C20—H20A···O5^i^	0.97	2.58	3.509 (12)	161.
C22—H22A···O4^iv^	0.97	2.58	3.530 (5)	165.
C22—H22B···O6a^i^	0.97	2.54	3.342 (9)	140.

Symmetry codes: (iii) −*x*+1, −*y*+2, −*z*+1; (i) *x*−1, *y*, *z*; (iv) −*x*+1, −*y*+1, −*z*+1.
